# Advances in pain management for older patients with cancer

**DOI:** 10.3332/ecancer.2019.980

**Published:** 2019-12-03

**Authors:** Georges El Hachem, Francisco Oliveira Rocha, Thierry Pepersack, Youssef Jounblat, Annie Drowart, Lissandra Dal Lago

**Affiliations:** 1 Department of Hematology and Medical Oncology, Saint George Hospital University Medical Center, University of Balamand, PO Box 166378, Ashrafieh, Beirut 1100 2807, Lebanon; 2 Medical Oncology Department, Institut Jules Bordet, L’Université Libre de Bruxelles (U.L.B), Brussels, Belgium; 3 Department of Hematology and Medical Oncology, Lebanese University, PO Box 6573/14, Badaro, Museum, Beirut, Lebanon; * Georges El Hachem and Francisco Oliveira Rocha contributed equally to writing this article.

**Keywords:** Geriatric patients, cancer, pain assessment, pain management

## Abstract

The population of older patients is growing with a rising prevalence of cancer diagnoses and cancer-related pain syndromes. Older patients are also vulnerable to misleading pain evaluations and under treatment with opioids. Barriers to the effective and safe management of analgesics include pain assessments and the complex management of the best analgesic choice and dose-titration while achieving the fewest side effects. In this review, we will provide an overview of the challenges present in assessment and treatment choices, along with practical tips for routine clinical practice.

## Introduction

Cancer risk increases with age, and a rapidly growing older population will increase the demand for cancer care. The diagnosis and treatment of cancer and cancer-related symptoms in older age groups are often complicated by other medical conditions. Despite therapeutic advances in analgesia, cancer-related pain remains an important and often unresolved problem [1]. At least 50% of cancer patients present with pain, with one-third being graded as moderate to severe. The highest prevalence of pain concerns is within the metastatic setting, where the goal of any cancer treatment is palliative [2].

Nowadays, geriatric oncology has become an independent speciality that deals with a population that is quite different from the patients included in most published clinical trials. Currently, information on the particular management of cancer-associated pain has to be extrapolated from younger patients [3]. There is a lack of randomised clinical trials studying cancer-related pain management, which have been performed exclusively in older adults [4]. In nursing home residents, 7 out of 10 geriatric patients are burdened with non-oncologic pain [5]. In the geriatric frail population, there is a high prevalence of cognitive impairment, multiple comorbidities and malnutrition and potentially inappropriate medications that may challenge pain management [6].

This article provides an overview on the specific aspects of pain assessment and management in geriatric oncology patients, with special attention paid to analgesic choices and drug interactions.

## Geriatric oncology specificity

The amplitude of pain and the way it affects frail older adults with cancer is substantial. Ageing leads to an alteration in the pharmacokinetic and pharmacodynamic profiles, including a narrow therapeutic index [7], delayed drug elimination due to hepatic and renal insufficiency and reduced receptor sites for drug binding and reduced volume of distribution [8, 9]. Although geriatric oncology patients have the particularity of a different physiological reserve and usually more comorbidities, they share the same cancer-related symptoms as the younger population.

Frail older patients might express their symptoms in different ways when compared to younger ones [10]. Cataldo *et al* [11] demonstrated in a cross-sectional study that older patients reported significantly fewer pain symptoms than their younger counterparts. Nipp *et al* [4] performed a secondary data analysis from a randomised two-by-two factorial trial in order to determine whether pain medications used in older patients were associated with better pain control. The population consisted of frail individuals aged 65 or older who were randomised to receive care in a geriatric inpatient unit, outpatient clinic, both or neither. Ninety-nine individuals with cancer were included, of whom 44 received a geriatric evaluation and were treated in a management unit care (GEMU) and 55 were treated with standard care procedures. GEMU patients (from inpatient or outpatient clinics) had a higher number of interventions as compared with those in usual care, mainly in the form of psychiatry, endocrinology, psychology and occupational and physical therapy. Besides there being no significant difference in the pain medication used between the intervention and usual care groups, there was significantly better pain control in the individuals of the GEMUs, suggesting that this might be related to an interdisciplinary approach [4].

In dementia, changes to pain perception and processing appear to vary in direction and quality depending on the type of neuropathology, pain and the severity of dementia [12]. Studies to date have indicated that pain is no less frequent or intense when experienced by these patients, despite changes in their ability to communicate [12]. Current evidence also suggests that pain perception and pain processing are not reduced in Alzheimer’s disease [13], as it does not necessarily affect the somatosensory cortex where the brain perceives stimuli to be painful [7].

From this perspective, palliative care specialists and medical oncologists should be aware of certain geriatric specificities in order to adapt pain cancer evaluation and management. It has been demonstrated that geriatric patients accept pain as an inevitable part of cancer [14] and often report concerns about pain treatments and side effects. Physicians usually prescribe analgesics differently for this population, as they tend to care more about side effects. However, it has been demonstrated that uncontrolled pain might worsen a patient’s frailty and participates in the deterioration of the performance status and quality of life of geriatric oncology patients [15].

## Pain assessment in the geriatric population

The complexity of cancer-related pain and its symptoms affect many aspects of a geriatric patient’s life and proxies. Pain can heavily affect their levels of physical activity, social interactions, sexuality and emotional and psychological status’ [16]. A thorough anamnesis, including details of the underlying malignant disease and received treatments as well as the impact of pain on their quality of life should be taken [17]. Patients in remission for cancer may also still have pain, such as phantom pain, pain from irradiated sites and chemotherapy or surgery-related neuropathy [18].

Pain assessments can be very challenging in the presence of cognitive impairment, behavioural changes and communication barriers, where a standard simple verbal rating scale and visual analogue scale may not be useful [19, 20–22]. Indeed, older patients tend to hide symptoms of pain and avoid expressing the need for relief. Some patients might believe that pain reflects the severity of a cancerous disease. Others are afraid of being a source of bother to their families or healthcare providers, while some think that the side effects of analgesics might worsen their medical condition [19]. While assessing pain in the older population, clinicians should also assess their concurrent comorbidities. Furthermore, the ability to perceive pain due to age-related loss of sensory neurons might also affect pain assessments in older age groups [8, 23].

The self-reporting of pain is often unattainable in geriatric patients with cognitive impairment due to difficulties in the comprehension of commonly used pain assessment tools [24–28]. Lukas *et al* [25] found that while a numerical rating scale could be used by 75% of older adults with mild cognitive impairment, only 57% of patients with moderate impairment and none of the severely impaired patients could utilise the tool. Despite a considerable number of pain assessment tools being available for use in the older, cognitively impaired population, there is limited evidence regarding their reliability, validity and clinical utility [29]. In patients with cognitive impairment, acute and breakthrough pain are usually associated with grimacing and sympathetic hyperactivity, such as tachycardia, tachypnoea, hypertension and diaphoresis, whereas such findings are often absent in chronic pain. The latter is usually associated with vegetative signs, including lassitude, sleep disturbance and anorexia (see Table 1). Careful monitoring of such manifestations might be very helpful in pain detection and assessment [30]. Also, the assessment of discomfort in dementia protocol can be very helpful in such situations. It is based on the assumption that behaviours associated with dementia are symptoms of unmet physiologic and/or non-physiologic needs. It takes into consideration the behavioural symptoms that are mentioned in Table 1, along with a careful assessment of the vital signs and a review of systems with a physical examination [31]. The checklist of nonverbal pain indicators (CNPI) is also based on the assessment of vocal, verbal and facial expressions, along with bracing, restless and rubbing movements at rest and upon movement. A score ranging from 0 to 10 can be calculated and used to assess the pain and response to analgesics [32, 33]. Other scales based on the same principles, such as the nursing assistant-administered instrument to assess pain in demented individual (NOPPAIN), the pain assessment scale for seniors with severe dementia (PACSLAC) and the pain assessment in advanced dementia scale (PAINNAD), can also be used in the pain assessments and evaluation of this population [34].

Herr *et al* [35] suggested that for non-communicating patients, an empiric analgesic trial should be initiated if there are pathologic conditions or procedures that are likely to cause pain, or if pain behaviours continue after attention has been paid to basic needs and comfort measures.

They suggested an analgesic trial and titration appropriate to the estimated intensity of pain based on a patient’s pathology and analgesic history be provided as follows: for mild to moderate pain, a non-opioid analgesic may be given initially (e.g., paracetamol every 4 hours for 24 hours), if behaviours improve, assume pain was the cause and continue with the analgesic adding appropriate non-pharmacologic interventions. If behaviours continue, consider giving a single low dose, short-acting opioid (e.g., hydrocodone, oxycodone or morphine) and observe the effects. If there is no change in behaviour, titrate the dose upward by 25%–50% and observe the effects. Continue to titrate upward until a therapeutic effect is seen, bothersome side effects occur or no benefit is determined. It may be appropriate to start the analgesic trial with an opioid for conditions in which moderate to severe pain is expected. Explore other potential causes if the behaviours continue after a reasonable analgesic trial. This analgesic titration example is conservative, and although strategies for safe titration should be followed, more aggressive approaches may be needed [36].

## Pain management

Despite the fact that a substantial amount of literature has been devoted to the topic of pain in cancer patients, the management of cancer pain in the geriatric population is still extrapolated from the guidelines of younger adult patients [37].

### Non-pharmacological approach

There is no validated standard non-pharmacological approach published for pain control in the cancer population [38]. Certainly, a complementary approach towards pain control in older patients requires multidisciplinary management. Among older adults with chronic non-cancer related pain, there is evidence that the addition of a non-pharmacological approach might enhance the pharmacological benefit, for example, with the use of cognitive and behavioural techniques for short-term pain relief [39]. Psychological support has been demonstrated to modify the subjective perception of the pain experience in older adults [40].

Depression is highly prevalent in cancer, and especially among people with chronic pain [41, 42]. It is underreported in older patients where it should always be suspected and managed adequately [43].

Despite being less studied in older populations, palliative antalgic radiation therapy is being used as an efficient treatment for pain control. However, it must be considered that palliative radiation therapy, even with limited fields, can be associated with higher rates of myelotoxicity in the geriatric population, as they dispose of a lower bone marrow reserve as compared with younger populations [44, 45].

### Pharmacological management

Unfortunately, numerous studies have documented that older patients are often undertreated for pain, with patterns including lower doses of analgesics and the use of only non-opioid pain-reliefs [46]. Guidelines from the National Comprehensive Cancer Network (NCCN), European Society of Medical Oncology (ESMO) [3] and the American Geriatric Society (AGS) recommend the use of the WHO sequential three-step analgesic ladder from non-opioids to weak opioids to strong opioids [47]. This is the same pharmacologic approach as for younger adults, but with the direction to ‘start low and go slow’ as the general rule of opioid titration. This strategy helps to compensate for possible concerns such as diminished drug metabolism and careful titration to effect [48, 49]. No research confirms that weight should be used in determining the starting dose [50, 51].

### Non-opioids, adjuvants and bone antiresorptive therapies

Paracetamol is the first current option associated with less side effects in older adults, provided that maximal dose is respected. However, it is rarely sufficient for the control of cancer-related pain as monotherapy, but can be associated with adjuvant treatments [52].

According to Cochrane Systematic Reviews [53], there is no enough evidence to support the use of oral non-steroidal anti-inflammatory drugs (NSAIDs) alone or in combination with another ladder of opioid medications to relieve cancer-related pain in adults. Furthermore, NSAIDs can more frequently result in undesirable side effects in older patients, mainly in the form of peptic ulcer disease and renal failure, and therefore, NSAIDS are considered an inappropriate prescription for this population [54].

Adjuvant treatments might diminish opioid requirements by increasing the efficacy of other analgesics and be more effective for specific pain, such as neuropathic pain.

Glucocorticoids are the most potent anti-inflammatory drug and are able to reduce cancer pain by inhibiting cytokines and prostaglandin as well as reducing vascular permeability. Glucocorticoids are graded as a moderate analgesic with higher rates of toxicity, particularly over 8 weeks [55]. Nonetheless, steroids are associated with many side effects in the geriatric population, including worsening of frailty due to proximal myopathy, an increased risk of falls, osteoporosis and associated fractures, infections, aggravation of other cardiovascular-associated comorbidities, insomnia, confusion and delirium [52, 56, 57]. However, they might be considered for short periods to avoid pain flares after radiotherapy and are a good option for end of life situations [58].

Antidepressants used in the treatment of chronic neuropathic pain include tricyclic antidepressants, selective serotonin reuptake inhibitors and selective noradrenaline reuptake inhibitors, all of which have increased side effects in older adults. As such, the choice should be based on the side effect profile of each drug. Tricyclic antidepressants are better avoided in this population due to anticholinergic effects and cardiac toxicity [59]. Anticonvulsants, such as pregabalin and gabapentin, have a better safety profile when used as an adjuvant treatment for persistent neuropathic pain in older adults [23].

The use of bone antiresorptive therapies, such as bisphosphonates and denosumab, is not recommended as immediate strong analgesics or to replace radiation therapy, but their addition for pain treatment is beneficial when bone pain is widespread and difficult to localise or in cases of bone pain recurrence after radiotherapy. In older patients with bone metastases that are candidates for bisphosphonates, particular attention should be paid to its potential for renal toxicity with renal monitoring. The product label advocates stepwise dose reductions when baseline creatinine clearance is 30–60 ml/minute, and zoledronic acid is not recommended in patients with severe renal deterioration [60]. Denosumab has been demonstrated to prevent the progression of pain severity and pain interference in HrQOL in the integrated analysis of patient-reported outcomes and analgesic use from three randomised trials of denosumab and zoledronic acid [61].

### Mild opioids

These agents are not considered the best option, as they are associated with an increased risk of confusion and alterations in the mental status of older patients. A systematic review of six observational studies demonstrated that the use of tramadol or meperidine was associated with an increased risk of delirium, whereas the use of morphine, fentanyl, oxycodone and codeine was not when compared to non-opioid medications [62].

### Strong opioids

Clinician’s experience, patient’s previous experiences and availability of a drug from local pharmacies are determining factors in opioid selection for older adults.

Even though the use of opioid analgesics for moderate to severe pain is becoming increasingly accepted [23], published data still indicate an underuse of opioids in the older population [63–65]. This under-prescription might be explained by the potential associated side effects. Controversially, one meta-analysis has demonstrated that old age is associated with a low risk for opioids abuse and misuse [66]. Upon initiation, patients should be monitored with screening tools to assess the risk of the likelihood of opioid misuse. Such monitoring starts with opioid risk tools scores and might extend to the use of urine toxicology screens on a periodic basis [67]. Moreover, physicians should assess and guide patients and caregivers on the safe storage of the drug, given the risks for drug mixing, overdose and using opioids for a purpose other than pain reduction.

As older patients might have an increased sensitivity to opioids, drug–drug and drug–disease interactions should be monitored prior to the initiation of therapy [68]. As ageing can lead to a higher incidence of opioid-related side effects, clinicians must initiate them at the lowest possible dose and titrate upwards based on tolerability and efficacy. The presence of multiple comorbidities, polypharmacy and physiologic vulnerability can worsen adverse events where they exist [69, 70]. It has been proposed to initiate opioids at 25%–50% of the adult recommended starting dose in patients older than 70 years in order to minimise side effects [71]. Chou *et al* [72] suggested biweekly monitoring during the initiation and dose-titration phase of treatment, and whenever the goals are not reached, the drug should be tapered and discontinued.

There is a well-established short-term efficacy for strong opioid therapy among older adults (≤12 weeks) [73]. In a retrospective study, Reid *et al* [65] evaluated the opioid prescriptions for older patients suffering from chronic musculoskeletal pain. One-hundred thirty-three patients (mean age 82) were newly started on opioid therapy. This attitude led to a decrease in pain for 66% of the patients. However, opioids were discontinued in 48% of patients, mostly as a result of poorly tolerated side effects, including constipation, urinary retention, changes to mental status and nausea [65].

Previous studies have demonstrated that pharmacokinetics and pharmacodynamics depend on the glomerular filtration rate and liver excretion capacity of the patient. As the drug availability and distribution changes in older patients, the onset and duration of action can be affected. Moreover, the *mu* receptor concentration has not been evaluated in the geriatric population, and this may be considered to possibly affect opioid activity [74].

Morphine is the most studied opioid analgesic and must be cautiously prescribed in cases of liver and renal failure. All long-acting opioids cannot be open or crushed before oral administration. If patients are unable to swallow, fentanyl or buprenorphine transdermal patches might be a better option. Hydromorphone and methadone are usually prescribed when high doses of opioids are required, or if there is an appearance of severe side effects with morphine. The long and variable half-life observed with methadone treatment requires hospitalisation for the careful titration and monitoring of side effects, especially in the risk of respiratory depression [75, 76]. Further recommendations applicable to older patients when selecting opioids in the geriatric population are available in Table 2 [77].

The starting oral doses of opioids depends mainly on the pain intensity, patient’s weight, patient’s general condition and previous treatment exposure. The ESMO, ASCO and NCCN guidelines included older age in their special population categories where opioids must be prescribed with caution [3, 37, 48, 49].

Healthcare workers and patients’ families must know that there is no dose limitation for opioids, with the correct dose being the dose relieving the patient’s symptoms without adding side effects. When patients are receiving strong opioids without achieving the analgesic effect or when pain control is achieved at the expense of important side effects, it is suggested to apply the theory of ‘opioid rotation’. This means that clinicians may change the opioid sub-class or keep the same molecule with a different route of administration [78, 79].

Table 3 summarises the analgesic drugs and highlights the pros and cons of each agent in the prescription for older patients with cancer.

## An attempt of recommendations for geriatric patients

- The heterogeneity of the ageing process and health status’ do not allow for the deliverance of general guidelines on the management of pain in geriatrics, *an individualised approach* should be proposed according to the geriatric characteristics.
- Take into account the elderly persons frailty in terms of the importance of *a careful and appropriate anamnesis* (given the fact that complaints are sometimes not formulated, misunderstood, trivialised or in the presence of cognitive disorders).
- Consider that *pain is a medical emergency* since it is often accompanied by rapid functional decline and a reduction in quality of life.
- *Integrate pain screening and management systematically* into the comprehensive geriatric assessment.
- For non-communicating patients, an* empiric analgesic trial* should be initiated where there are pathologic conditions or procedures that are likely to cause pain or if pain behaviours continue after attention has been paid to basic needs and comfort measures
- Take into account the frequent presence of poly-pathology, which sometimes makes it difficult to understand the underlying problems causing the painful complaints, often requiring a targeted approach towards the *identification of several potential aetiologies.*
- Account for geriatric characteristics, particularly with respect to *age-related pharmacokinetics and pharmacodynamics.*
- *Screen for potentially inappropriate prescriptions* (under-, mis- and over-prescription) in general, but also for pain medication (e.g., avoid tricyclics, suppress concomitant opioid prescriptions of the second and third step, do not forget the case of prescribing drugs for neuropathic pain and recognise side effects, etc.).

## Conclusion

Pain management of the geriatric population can present many challenges to clinicians. Uncontrolled pain has been associated with a poor quality of life and worse oncological outcomes. Close attention must be paid to this special population, ensuring a continuous knowledge of the high prevalence of polypharmacy, risk of drug interactions and physiological modifications affecting the analgesic’s metabolism. Careful monitoring of frail patients is necessary to effectively assess and manage pain while minimising the potential for adverse events.

## Conflicts of interest

The authors declare that they have no conflicts of interest.

## Funding statement

All of the authors declare that they had no access to funding support for this work.

## Authors’ contributions

Georges El Hachem and Francisco Oliveira Rocha contributed equally to writing this article. Youssef Jounblat also contributed to writing this article. Annie Drowart, Thierry Pepersack and Lissandra Dal Lago reviewed this article and contributed to the editing of the text.

## Figures and Tables

**Table 1.** Different signs and symptoms associated with pain in patients with cognitive impairment.

Facial expressions	Frowning, grimacing, distorted expression and rapid blinking
Verbalisations/vocalisations	Sighing, moaning, calling out, asking for help and verbal abuse
Body movements	Rigidity, tension, guarding, increased pacing/rocking, inactivity or motor restlessness
Changes in interpersonal interactions	Aggressive, resisting care, disruptive and withdrawn
Mental status change	Crying, sadness, increased confusion, irritability and distress
Physiological changes	Tachycardia, tachypnea, hypertension and diaphoresis and pupil dilatation

**Table 2.** Opioids’ safety in renal and hepatic dysfunctions.

Opioids in patients with renal failure			Opioids in patients with liver failure		
Not recommended	Use cautiously	Appears safe	Not recommended	Use cautiously	Appears safe
Meperidine	Morphine	Fentanyl	Meperidine	Morphine	Fentanyl
Codeine	Oxycodone	Methadone	Codeine	Oxycodone	
Propoxyphene		Hydromorphone	Propoxyphene	Hydromorphone	
		Buprenorphine	Methadone		

**Table 3.** Drugs for cancer pain treatment in older patients.

Drug	Geriatric considerations	Side-effects	Interactions	Mechanism/Initial dose and titration/formulations
**Tradonal** [80, 81]	• Metabolite accumulation in case of renal impairment. • Avoid utilisation in older patients at risk for seizures. • Risk of respiratory depression and sedation if associated with other CNS depressants (like benzodiazepines).	• Increased risk of delirium when compared with other opioids. • Risk of serotonin syndrome. • Drowsiness, constipation and nausea.	• Interaction with serotoninergic medication.	• *mu*-receptor agonist. • Titration: start with low dose 12.5–25 mg (immediate release). • Formulation: PO, IR, IV, SQ and IM.
**Buprenorphine** [82, 83]	• Good analgesic control when used as a single agent, to prevent poly-pharmacy. • Monitor closely at the starting dose and titration. • Can be used in case of renal failure. • Low addiction potential. • Anti-depressive and antianxiety effects.	• Cognitive function seems not affected by low TD dose. • Lower risk of nausea, vomiting and constipation. • Monitor for respiratory depression and dizziness in older patients (risk for falls/fractures). • TD patch: application-site pruritus, hypotension.		• Semisynthetic opioids *mu*-receptor agonists. • TD patch: start with a 5 mcg/hour every 3–4 days. • Formulation: IM, IV, PO, TD.
**Fentanyl** [84]	• The TD administration should be avoided in cachectic cancer patients. • TD administration is an option to oral opioid intolerance. • Oral transmucosal administration can provide effective and rapid onset of pain relief. • Not recommended if severe renal or hepatic impairment.	• Constipation, nausea, vomiting, confusion, dizziness, sedation, dyspnoea and erythema at application-site. • Serotonin syndrome.	• Absorption increases in case of fever or cachexia/severe sarcopenia. • Metabolised by the cytochrome P450 system.	• Synthetic opioid, *mu*-receptor agonist. • It should be avoided as a frontline option in opioids-naïve patients. • Initial dose is determined by titration using immediate-release opioid for few days. • Lowest patch dose: start at 12 mcg/hour. • Maximum effect of TD patch is obtained after 12–24 hours. • TD administration. • Formulation: IV/TM/trans-mucosal.
Hydromorphone [62, 74]	• Patients who present with dysphagia can benefit from the liquid form, available for hydromorphone, oxycodone and morphine. • Efficacy in patients with sleep disturbance related to cancer pain. • Dose reduction recommended in case of renal impairment.	• Decreased risk of delirium compared with other opioids.	• Metabolism to apparently inactive metabolites is an advantage over morphine in older adults with renal or hepatic insufficiency. • Accumulation of a neurotoxic (excitatory) metabolite may become a concern in older adults with severe renal insufficiency.	• Starting dose by 1–2 mg every 3–4 hours and determine the 24-hour dose requirement after 3–7 days. • Formulation: oral/IV.
**Methadone** [85]	• Lack of data about the utilisation of methadone in older cancer population. • Not recommended as a first-line opioid. • Can be effective in cases of neuropathic pain.	• Risk of apnoea at high doses. • Increased risk of cardiac arrhythmias by QT interval prolongation. • Always prescribed in patients who are hospitalised.	• Metabolised by the cytochrome P450 system: use with caution in case of poly-pharmacy. • Risk of overdose (accumulation) during initial titration.	• Starting dose: 1–2mg q3–4 hours. • Increase daily dose by 1–2 mg after 7 or more days. • Further increases in a daily dose of 1–2 mg should be made no more frequently than once every 7 days. • Extremely long half-life. • Formulation: SC/PO.
**Morphine** [84]	• Patients with lower volumes of distribution can have a higher peak plasma levels. • Patients with renal failure might have toxic reactions due to the accumulation of metabolites. • Increased potential risk for falls and fractures. • Good option for treatment of neuropathic pain, with or without adjuvants.	• Side effects: nausea, vomiting, respiratory depression, dizziness and constipation. • In case of confusion, vesical retention and fecaloma to be excluded.	• Metabolised in the liver. • Renal excretion and metabolites accumulation lead to neurotoxicity.	• Starting dose at 50% of the adult’s dose. • Titration:Low dose starting: 5 mg every 4 hours, steep increase by 30%–50% per day. • Determine 24-hour dose requirement after 3–7 days. • Use of laxatives is preconised • Formulations: IV/IR/PO/SC.
**Oxycodone** [84]	• A great choice for older patients due to the absence of toxic metabolites and short half-lives. • Low dose associated with less alteration of cognitive function. • Can be useful for neuropathic pain treatment.	• Like fentanyl and methadone, can be considered as a less constipating opioid. • Headache, drowsiness, dizziness, nausea and vomiting.	• No toxic metabolites. • Both hepatic and renal metabolism.	• Start with 2.5–5 mg orally every 4 hours as needed. • After 3–7 days, determine 24-hour dose requirement. • Immediate-release onset of action 15 minutes. • Extended-release onset of action 1 hour. • Formulation: PO.
**Gabapentin and pregabalin** [86]	• Effective for treatment of neuropathic pain and as adjuvant treatment to other painful conditions.	• Sedation and dizziness might limit the use in older adults.	• Clearance highly dependent on renal function.	• Gabapentin: start with 100 mg/day at bedtime. • Pregabalin: start with 25–50 mg at bedtime. • Gradual titration over weeks: • Formulation: PO.
**Duloxetine and venlafaxine** [86–88]	• Effective adjuvant in the treatment of neuropathic pain, including chemotherapy-induced.	• Potential to cause or exacerbate syndrome of inappropriate antidiuretic hormone secretion (SIADH) or hyponatremia in older adults.	• To avoid in patients with severe renal impairment. • Fewer cardiovascular and anticholinergic effects than tricyclic antidepressants.	• Gradual titration over 2–4 weeks: • Duloxetine: start with 20–30 mg/day. • Venlafaxine: start with 37.5 mg orally daily. • Formulation: PO.

Abbreviations: CNS, central nervous system; PO, per os; IR, intra-rectal; IV, intravenous; SQ, sub-cutaneous; IM, intramuscular; mg, milligram; TD, transdermal; mcg, microgram; d, day
